# Supplementary material for: Experimental assessment of AI-based interactome mapping
Source: Nat Commun. 2026 Apr 4;17:4894. doi: 10.1038/s41467-026-70942-x (PMC13230783; doi:10.1038/s41467-026-70942-x)
Supplement: Supplementary file 1 — Supplementary Information [file 41467_2026_70942_MOESM1_ESM.pdf]

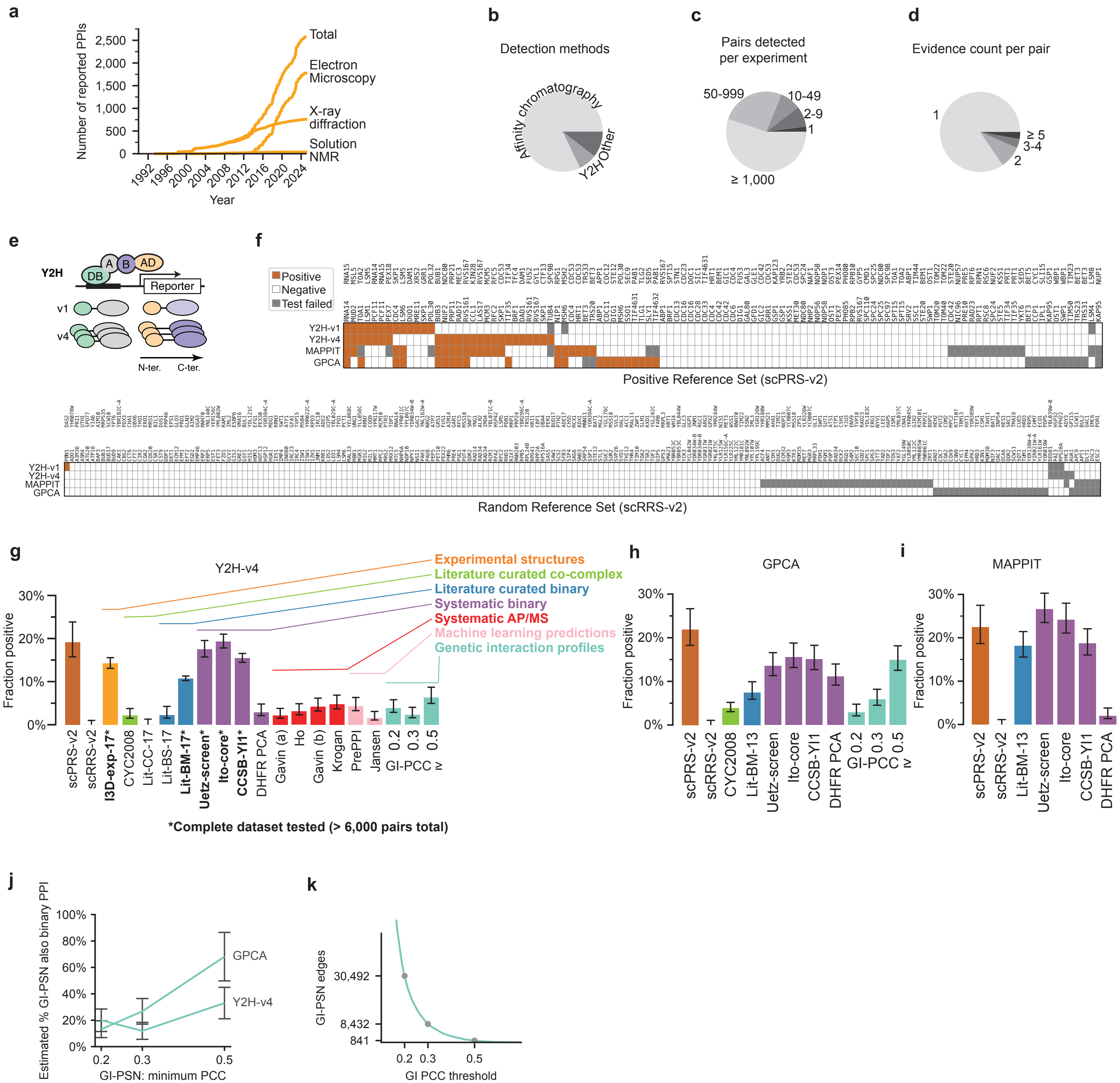
a
Number of reported PPIs
2,500
2,000
1,500
1,000
500
0
1992
1996
2000
2004
2008
2012
2016
2020
2024
Year
Total
Electron Microscopy
X-ray diffraction
Solution NMR
b
Detection methods
Affinity chromatography
Y2H
Other
c
Pairs detected per experiment
50-999
10-49
2-9
1
≥ 1,000
d
Evidence count per pair
1
≥ 5
3-4
2
e
Y2H
A
B
AD
DB
Reporter
v1
v4
N-ter.
C-ter.
f
Positive
Negative
Test failed
Y2H-v1
Y2H-v4
MAPPIT
GPCA
Positive Reference Set (scPRS-v2)
Random Reference Set (scRRS-v2)
g
Y2H-v4
Experimental structures
Literature curated co-complex
Literature curated binary
Systematic binary
Systematic AP/MS
Machine learning predictions
Genetic interaction profiles
Fraction positive
30%
20%
10%
0%
scPRS-v2
scRRS-v2
I3D-exp-17*
CYC2008
Lit-CC-17
Lit-BS-17
Lit-BM-17*
Uetz-screen*
Ito-core*
CCSB-YI1*
DHFR PCA
Gavin (a)
Ho
Gavin (b)
Krogan
PrePPI
Jansen
GI-PCC ≥
0.2
0.3
0.5
*Complete dataset tested (> 6,000 pairs total)
h
GPCA
Fraction positive
30%
20%
10%
0%
scPRS-v2
scRRS-v2
CYC2008
Lit-BM-13
Uetz-screen
Ito-core
CCSB-YI1
DHFR PCA
GI-PCC ≥
0.2
0.3
0.5
i
MAPPIT
Fraction positive
30%
20%
10%
0%
scPRS-v2
scRRS-v2
Lit-BM-13
Uetz-screen
Ito-core
CCSB-YI1
DHFR PCA
j
Estimated % GI-PSN also binary PPI
100%
80%
60%
40%
20%
0%
0.2
0.3
0.5
GI-PSN: minimum PCC
GPCA
Y2H-v4
k
GI-PSN edges
30,492
8,432
841
0.2
0.3
0.5
GI PCC threshold

Supplementary Fig. 1 | Tests of existing interaction datasets. a, Growth of PPIs with experimental structures over time. **b-d**, Evidence for those published interacting protein pairs, split by (**b**) interaction detection assay, (**c**) experiment size, and (**d**) evidence count per pair. **e,** Schematic showing the differences between Y2H v1 and v4. **f,** Benchmarking four binary PPI assays, using positive and random reference sets (scPRS-v2 and scRRS-v2). **g-i,** Extended version of Fig. 1f-h –Experimental validation of various interaction datasets recovered in (**g**) Y2H-v4, (**h**) GPCA, or (**i**) MAPPIT – showing all tested datasets. In Fig. 1g-i, AP/MS corresponds to Krogan; ML to PrePPI; GI-PSN to PCC $\geq$ 0.3; and Y2H-union-08 is the full union of the 3 constituent datasets, since every pair was tested, this avoids double counting of pairs in multiple source datasets. **j**, Using the Y2H-v4 and GPCA recovery results to estimate the fraction of GI-PSN pairs that correspond to a binary PPI. **k**, Size of the GI-PSN as a function of the PCC threshold used to define the edges.

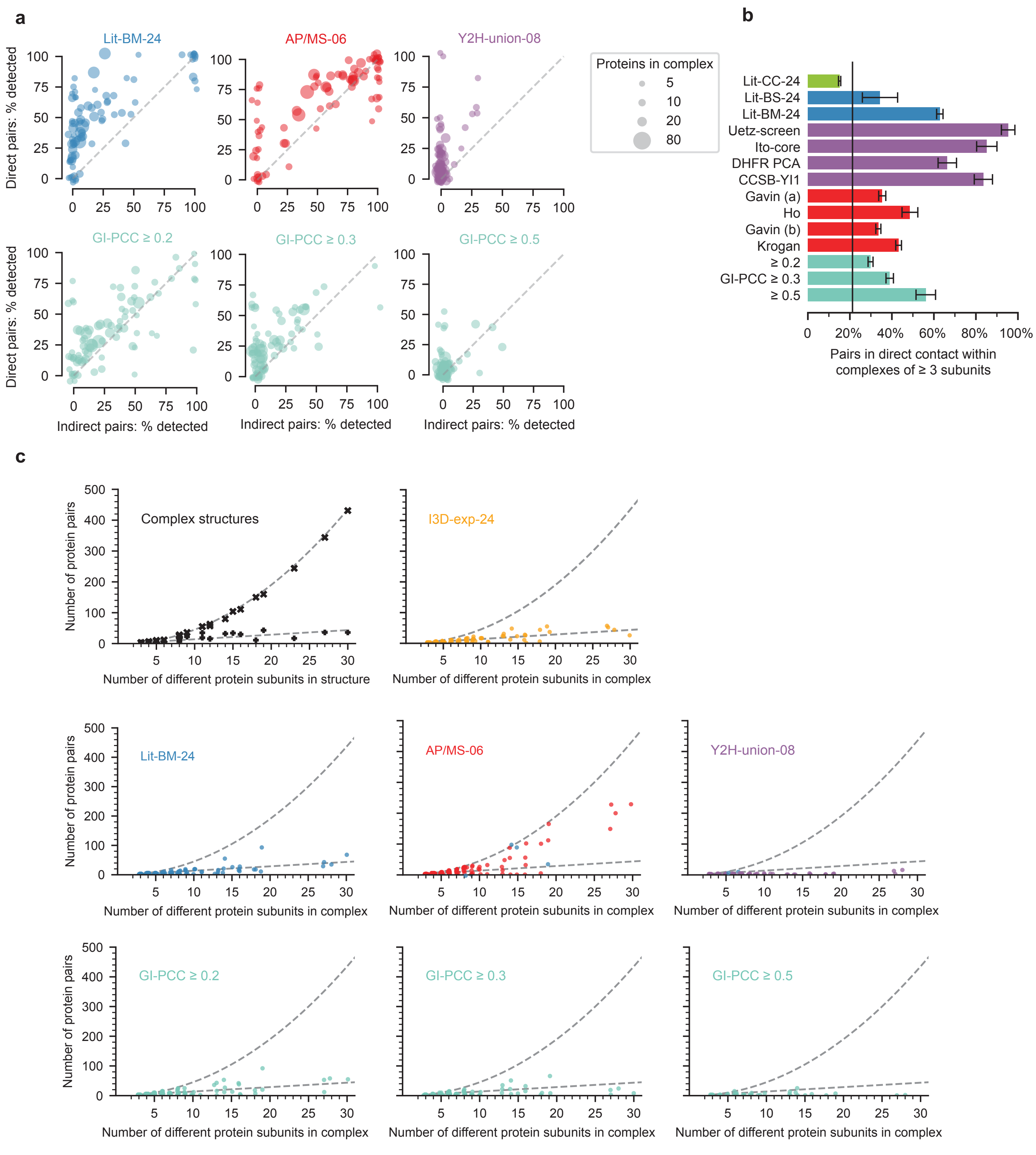

a
Lit-BM-24
AP/MS-06
Y2H-union-08
Proteins in complex
5
10
20
80
Direct pairs: % detected
GI-PCC ≥ 0.2
GI-PCC ≥ 0.3
GI-PCC ≥ 0.5
Indirect pairs: % detected
b
Lit-CC-24
Lit-BS-24
Lit-BM-24
Uetz-screen
Ito-core
DHFR PCA
CCSB-YI1
Gavin (a)
Ho
Gavin (b)
Krogan
≥ 0.2
GI-PCC ≥ 0.3
≥ 0.5
Pairs in direct contact within complexes of ≥ 3 subunits
c
Complex structures
I3D-exp-24
Number of protein pairs
Number of different protein subunits in structure
Number of different protein subunits in complex
Lit-BM-24
AP/MS-06
Y2H-union-08
GI-PCC ≥ 0.2
GI-PCC ≥ 0.3
GI-PCC ≥ 0.5

Supplementary Fig. 2 | Many datasets contain large numbers of indirect associations. a, Recovery of protein pairs in direct contact and pairs not in direct contact within the corresponding 3D structures by different biophysical and functional networks. Each point is a separate protein complex with at least 5 distinct protein subunits. **b**, The fraction of directly contacting interactions, taking all reported pairs where both proteins are in a protein complex structure with at least three subunits, for different datasets. The vertical line denotes the total across all protein complex structures with at least three subunits. Error bars are standard error of proportion**. c**, Top-left panel: the number of either direct (+) or both direct and indirect (x) PPIs in the single largest experimental structure for each protein complex. The curved dashed line corresponds to the total possible pairwise combinations of different proteins per complex; the straight dashed line is a linear regression using the direct PPIs. All other panels: the number of PPIs in each of the five networks within each complex, against complex size. The dashed lines from the top-left panel are reproduced on each panel.

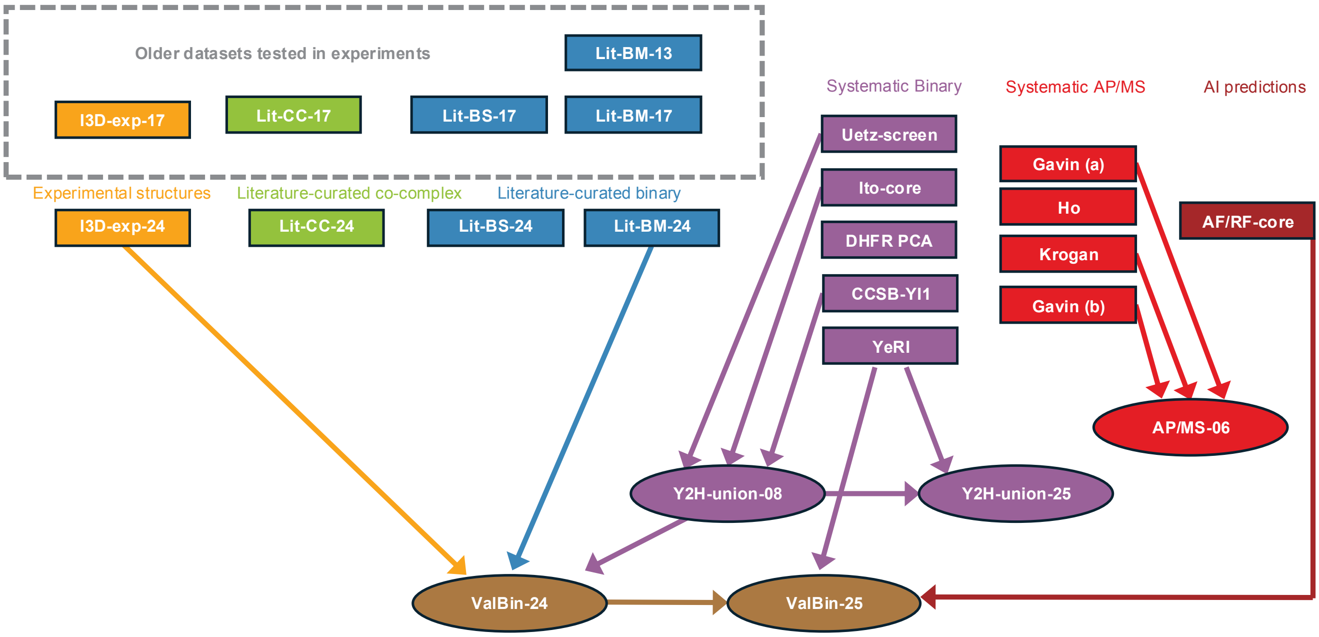
Older datasets tested in experiments
Lit-BM-13
I3D-exp-17
Lit-CC-17
Lit-BS-17
Lit-BM-17
Experimental structures
Literature-curated co-complex
Literature-curated binary
I3D-exp-24
Lit-CC-24
Lit-BS-24
Lit-BM-24
Systematic Binary
Uetz-screen
Ito-core
DHFR PCA
CCSB-YI1
YeRI
Systematic AP/MS
Gavin (a)
Ho
Krogan
Gavin (b)
AI predictions
AF/RF-core
AP/MS-06
Y2H-union-08
Y2H-union-25
ValBin-24
ValBin-25

Supplementary Fig. 3 | Schematic of the relationships between yeast datasets used in this study. a, Datasets connected by arrows denote the constituents of composite datasets. Dataset names ending in numbers refer to the year of generation.

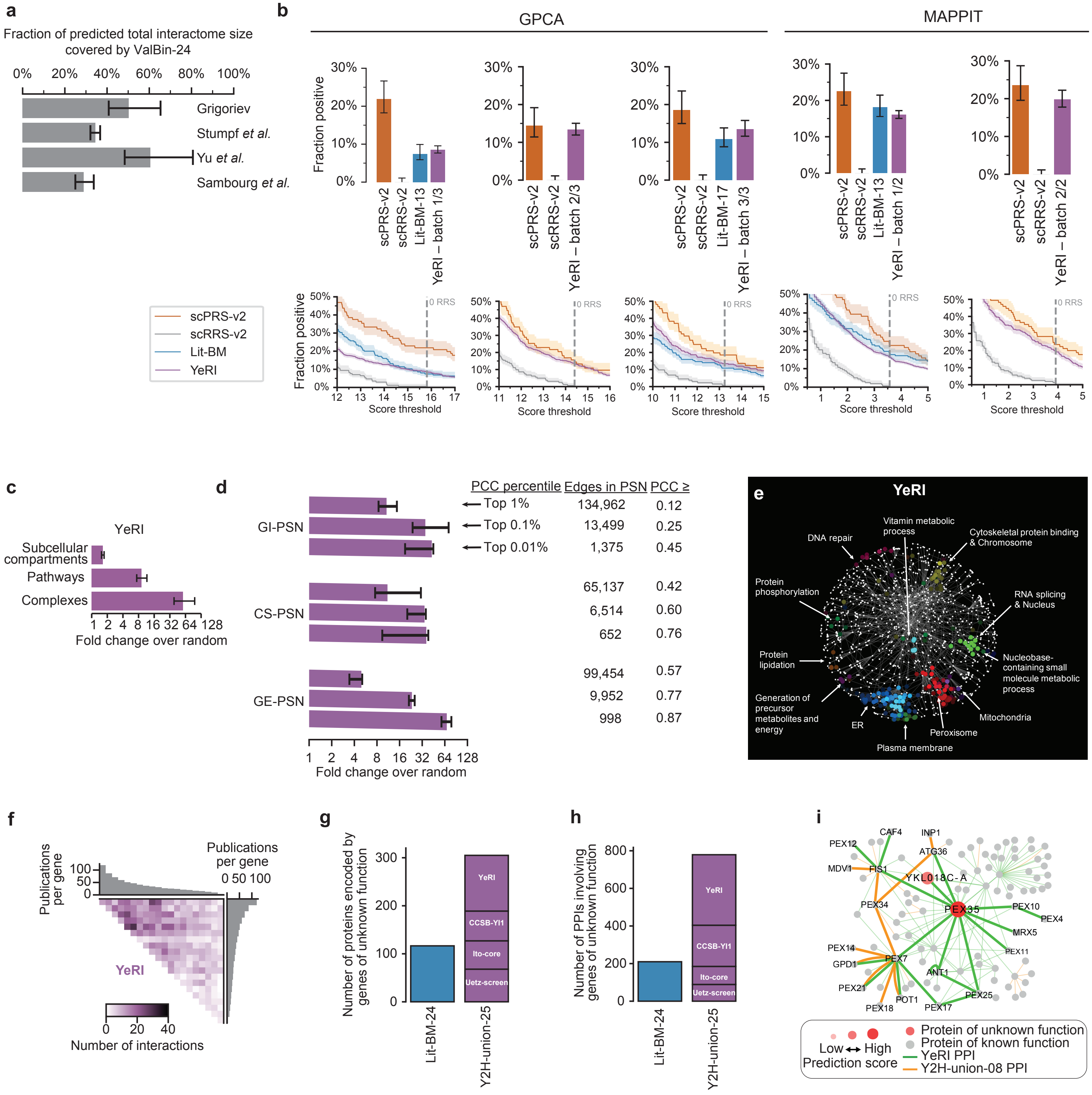

a
Fraction of predicted total interactome size covered by ValBin-24
0% 20% 40% 60% 80% 100%
Grigoriev
Stumpf et al.
Yu et al.
Sambourg et al.
b
GPCA
MAPPIT
Fraction positive
30%
20%
10%
0%
scPRS-v2
scRRS-v2
Lit-BM-13
YeRI – batch 1/3
YeRI – batch 2/3
Lit-BM-17
YeRI – batch 3/3
YeRI – batch 1/2
YeRI – batch 2/2
scPRS-v2
scRRS-v2
Lit-BM
YeRI
50% 40% 30% 20% 10% 0%
0 RRS
Score threshold
c
YeRI
Subcellular compartments
Pathways
Complexes
1 2 4 8 16 32 64 128
Fold change over random
d
PCC percentile
Edges in PSN
PCC ≥
GI-PSN
Top 1% 134,962 0.12
Top 0.1% 13,499 0.25
Top 0.01% 1,375 0.45
CS-PSN
65,137 0.42
6,514 0.60
652 0.76
GE-PSN
99,454 0.57
9,952 0.77
998 0.87
1 2 4 8 16 32 64 128
Fold change over random
e
YeRI
DNA repair
Vitamin metabolic process
Cytoskeletal protein binding & Chromosome
Protein phosphorylation
RNA splicing & Nucleus
Protein lipidation
Nucleobase-containing small molecule metabolic process
Generation of precursor metabolites and energy
ER
Plasma membrane
Peroxisome
Mitochondria
f
Publications per gene
100 50 0
Publications per gene
0 50 100
YeRI
0 20 40
Number of interactions
g
Number of proteins encoded by genes of unknown function
300 200 100 0
YeRI
CCSB-YI1
Ito-core
Uetz-screen
Lit-BM-24
Y2H-union-25
h
Number of PPIs involving genes of unknown function
800 600 400 200 0
YeRI
CCSB-YI1
Ito-core
Uetz-screen
Lit-BM-24
Y2H-union-25
i
PEX12
CAF4
INP1
ATG36
MDV1
FIS1
YKL018C-A
PEX34
PEX35
PEX10
PEX4
MRX5
PEX11
PEX14
GPD1
PEX7
ANT1
PEX21
POT1
PEX18
PEX17
PEX25
Low ↔ High
Prediction score
Protein of unknown function
Protein of known function
YeRI PPI
Y2H-union-08 PPI

Supplementary Fig. 4 | An experimental reference map adds a large number of novel PPIs. **a**, Coverage of the yeast interactome by the union of I3D-exp-24, Lit-BM-24, and Y2H-union-08, based on four reported estimates of the total interactome size. Error bars correspond to the 95% confidence intervals of each estimate reported in the original publications. **b**, The results of different batches of experimental validations of YeRI and other datasets by GPCA and MAPPIT. In total, all pairs from YeRI were tested in both assays. Top-row: bar charts using the threshold calibrated to 0 scRRS-v2 positives; bottom-row: full titration across the assay output score, where vertical dashed line shows the score at 0 scRRS-v2 positives. Error bars and shaded bands are 68.3% Bayesian credible intervals. **c**, Enrichment of YeRI for interacting protein pairs in the same subcellular compartment, pathway, or protein complex, relative to degree-preserved random networks. Central values are relative to the mean, and error bars relative to the inner 68.3%, of the random networks. **d**, Enrichment of YeRI for interacting protein pairs whose genes are directly connected in functional PSNs, relative to degree-preserved random networks. Central values are relative to the mean, and error bars relative to the inner 68.3%, of the random networks. **e**, Network-based spatial enrichment analysis (SAFE) for YeRI. Clusters of genes enriched for GO terms in YeRI are highlighted. **f**, Heatmap of PPI density, within the proteome-by-proteome search space, ordered by the number of associated publications per gene, for YeRI. **g**, The number of proteins encoded by genes of unknown function with at least one interaction in Y2H-union-25 or Lit-BM. **h**, Number of PPIs involving proteins encoded by genes of unknown function in Lit-BM-24 or Y2H-union-25. **i**, PPI network of PEX35 and its first- and second-degree interactors. Named proteins are those annotated or predicted to have the GO terms related to “peroxisome importomer complex”.

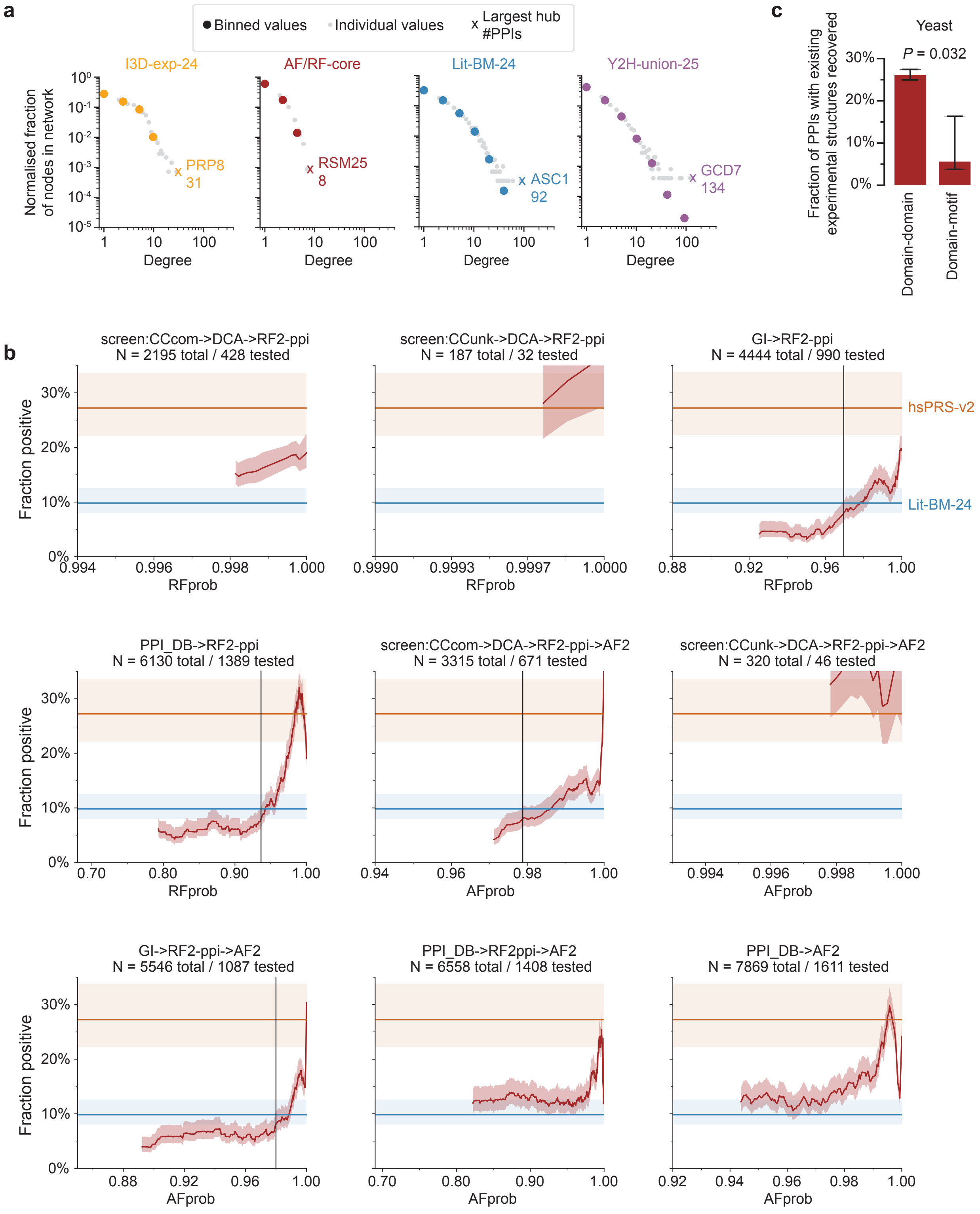
a
Binned values
Individual values
x Largest hub #PPIs
I3D-exp-24
AF/RF-core
Lit-BM-24
Y2H-union-25
Normalised fraction of nodes in network
PRP8
31
RSM25
8
ASC1
92
GCD7
134
Degree
c
Yeast
P = 0.032
Fraction of PPIs with existing experimental structures recovered
30%
20%
10%
0%
Domain-domain
Domain-motif
b
screen:CCcom->DCA->RF2-ppi
N = 2195 total / 428 tested
screen:CCunk->DCA->RF2-ppi
N = 187 total / 32 tested
GI->RF2-ppi
N = 4444 total / 990 tested
hsPRS-v2
Lit-BM-24
Fraction positive
RFprob
PPI_DB->RF2-ppi
N = 6130 total / 1389 tested
screen:CCcom->DCA->RF2-ppi->AF2
N = 3315 total / 671 tested
screen:CCunk->DCA->RF2-ppi->AF2
N = 320 total / 46 tested
AFprob
GI->RF2-ppi->AF2
N = 5546 total / 1087 tested
PPI_DB->RF2ppi->AF2
N = 6558 total / 1408 tested
PPI_DB->AF2
N = 7869 total / 1611 tested

Supplementary Fig. 5 | Testing AI-predicted interactome maps. a, Degree distributions of high-quality binary interactome maps. Large coloured points are binned values, gray points are unbinned values. **b**, Results of testing the human AF/RF dataset in Y2H-v4, as a sliding window across contact probability divided into 9 strategies[1]. Red: AF/RF, blue: Lit-BM-24, orange: hsPRS-v2. Error bands are 68.3% Bayesian credible intervals. Vertical lines indicate experimentally derived threshold where the mean of the sliding window crosses the lower bound of Lit-BM-24. **c**, Fraction of domain-domain and domain-motif mediated benchmark yeast PPI datasets that are present in AF/RF-core. Error bars are 68.3% Bayesian credible intervals. *P*-value calculated using one-tailed Fisher's exact test (1,250 domain-domain and 18 domain-motif PPIs).

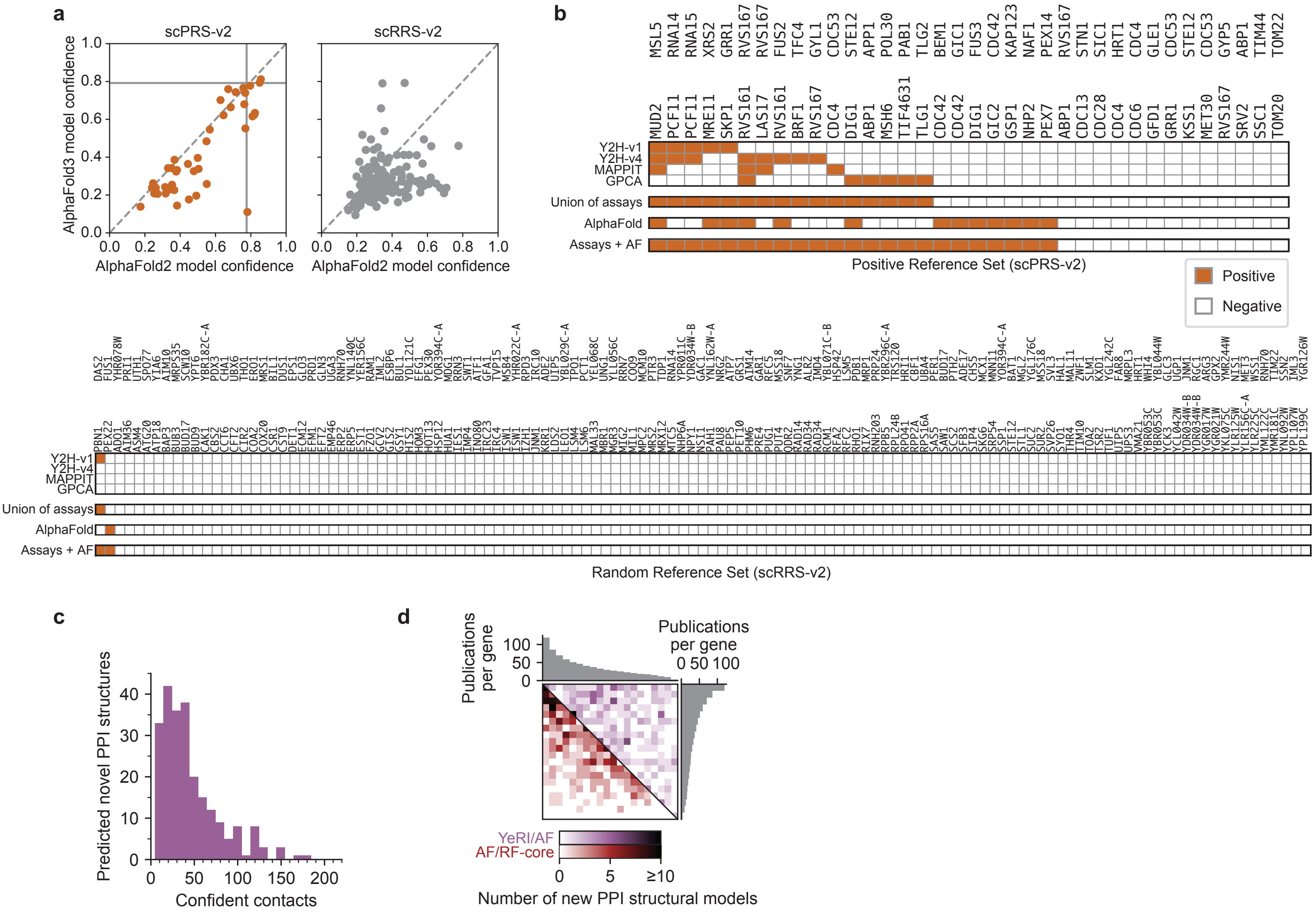
a
scPRS-v2
scRRS-v2
AlphaFold3 model confidence
1.0
0.8
0.6
0.4
0.2
0.0
0.0 0.2 0.4 0.6 0.8 1.0
AlphaFold2 model confidence
AlphaFold2 model confidence
b
MSL5 RNA14 RNA15 XRS2 GRR1 RVS167 RVS167 FUS2 TFC4 GYL1 CDC53 STE12 APP1 POL30 PAB1 TLG2 BEM1 GIC1 FUS3 CDC42 KAP123 NAF1 PEX14 RVS167 STN1 SIC1 HRT1 CDC4 GLE1 CDC53 STE12 CDC53 GYP5 ABP1 TIM44 TOM22
MUD2 PCF11 PCF11 MRE11 SKP1 RVS161 LAS17 RVS161 BRF1 RVS167 CDC4 DIG1 ABP1 MSH6 TIF4631 TLG1 CDC42 CDC42 DIG1 GIC2 GSP1 NHP2 PEX7 ABP1 CDC13 CDC28 CDC4 CDC6 GFD1 GRR1 KSS1 MET30 RVS167 SRV2 SSC1 TOM20
Y2H-v1
Y2H-v4
MAPPIT
GPCA
Union of assays
AlphaFold
Assays + AF
Positive Reference Set (scPRS-v2)
Positive
Negative
Y2H-v1
Y2H-v4
MAPPIT
GPCA
Union of assays
AlphaFold
Assays + AF
Random Reference Set (scRRS-v2)
c
Predicted novel PPI structures
40
30
20
10
0
0 50 100 150 200
Confident contacts
d
Publications per gene
100
50
0
Publications per gene
0 50 100
YeRI/AF
AF/RF-core
0 5 ≥10
Number of new PPI structural models

Supplementary Fig. 6 | Experimental PPI mapping combined with AlphaFold generates a high-resolution contactome map. **a**, Scatter plots of AlphaFold v2 vs v3 model confidence for scPRS-v2 (left) and scRRS-v2 (right). Gray lines represent the highest scoring scRRS-v2 pair. PPIs with existing experimental structures, including homologous proteins, are excluded. **b**, Benchmarking four binary PPI assays and AlphaFold2 using the CCC metric, using positive and random reference sets (scPRS-v2 and scRRS-v2). PPIs with existing experimental structures, including homologs, were excluded. Only pairs successfully tested with all methods are shown. **c**, Distribution of the number of confident residue-residue contacts, above a minimum threshold of five, for YeRI PPIs without previous structural models. **d**, A heatmap of PPI density of new structurally modeled PPIs from YeRI and AF/RF-core, within the proteome-by-proteome search space, ordered by the number of associated publications per gene.

Name	Description	# heterodimer edges	# proteins
I3D-exp-17	Experimental PPI structures	985	730
I3D-exp-24	Experimental PPI structures	2,576	1,408
Lit-CC-17	Literature-curated co-complex	70,492	5,600
Lit-CC-24	Literature-curated co-complex	158,843	5,835
Lit-BS-17	Literature-curated binary single evidence	13,736	4,712
Lit-BS-24	Literature-curated binary single evidence	15,090	4,671
Lit-BM-13	Literature-curated binary multiple evidence	3,778	2,259
Lit-BM-17	Literature-curated binary multiple evidence	4,260	2,519
Lit-BM-24	Literature-curated binary multiple evidence	6,309	3,011
Uetz-screen	Systematic binary	607	747
Ito-core	Systematic binary	738	766
DHFR PCA	Systematic binary	2,525	1,076
CCSB-YI1	Systematic binary	1,605	1,206
Gavin (a)	Systematic AP/MS	3,210	1,352
Ho	Systematic AP/MS	3,583	1,555
Krogan	Systematic AP/MS	7,055	2,670
Gavin (b)	Systematic AP/MS	6,531	1,430
YeRI	Systematic binary	1,880	1,346
AF/RF	AI predictions	1,499	1,566
AF/RF-core	AI predictions	969	1,177

Supplementary Table 1 | Description of biophysical datasets used in this study. Dataset names ending in numbers refer to the year of generation.

Name	Constituent datasets	# heterodimer edges	# proteins
Y2H-union-08	Uetz-screen + Ito-core + CCSB-YI1	2,613	1,884
Y2H-union-25	Y2H-union-08 + YeRI	4,307	2,505
ValBin-24	I3D-exp-24 + Lit-BM-24 + Y2H-union-08	9,817	3,713
ValBin-25	ValBin-24 + YeRI + AF/RF-core	11,602	4,056

Supplementary Table 2 | Composition of composite PPI datasets.

						Number of pairs attempted in:		
**Category**			**Dataset**	**Co-complex associations**	**Binary PPIs**	**Y2H-v4**	**GPCA**	**MAPPIT**
Experimentally detected	Co-complex	Proteome x proteome	Gavin (a)	3,210		200		
			Gavin (b)	6,531		200		
			CYC2008[a]	11,136		200	405	
			Krogan	7,059		200		
			Ho	3,584		200		
		Literature	Lit-CC-17	71,260		151		
	Binary	Proteome x proteome	Ito-core		816	738	199	199
			Uetz-screen		645	470	193	193
			CCSB-YI1		1,772	1,536	200	200
			Tarassov		2,761	199	199	199
		Structures	I3D-exp-17		1,787	1,231		
		Literature	Lit-BS-17		13,981	146	149	
			Lit-BM-13		4,115		584	168
			Lit-BM-17		4,623	4,128		
Predicted			Jansen	9,870		200		
			PrePPI[b]		30,184	200	198	
			AF/RF[c]		1,505	1,505		
PRS and RRS			scPRS-v2		108	108	108	108
			scRRS-v2		198*	198	198	198
YeRI			YeRI		1,910		1,910	1,910

[a] pairwise combinations of proteins within each complex
[b] high confidence subset
[c] tested across two Y2H-v4 experiments
*Negative control of random pairs of proteins

Supplementary Table 3 | Description of tested yeast datasets.

PSN	PCC threshold (top 1%)	Number of nodes	Number of edges
GI	0.12	5,328	134,972
CS	0.42	3,479	65,147
GE	0.57	3,832	99,454

Supplementary Table 4 | Numbers of genes and interactions in the top 1% percentile of the genetic profile similarity networks.

Assay version	DB vector	AD vector	DB yeast strain	AD yeast strain
1	pDEST-DB	pDEST-AD-*CYH2*	Y8930	Y8800
4	pDEST-DB-QZ212	pDEST-AD-QZ213	Y8930	Y8800

Supplementary Table 5 | Y2H assay version comparison.

**Name**	**pDEST-DB**	**pDEST-DB -QZ212**	**pDEST-AD -*CYH2***	**pDEST-AD -QZ213**
**Fusion partner (aa)**	Gal4-DB (1-147)	Gal4-DB (1-147)	Gal4-AD (768-881)	Gal4-AD (768-881)
**Fusion location**	N-terminus	N-terminus	N-terminus	N-terminus
**Yeast Promoter (nt)**	Truncated *ADH1* (-701 to +1)	Truncated *ADH1* (-410 to +1)	Truncated *ADH1* (-701 to +1)	Truncated *ADH1* (-410 to +1)
**Yeast replication of origin**	CEN	2μ	CEN	2μ
**Linker sequence between C-Term of Gal4 element and Gateway cloning site (aa)**	SRSNQ	PEFPS	GGSNQ	ICMAYPYDVPD YASLGGHMAM EAPS
**Yeast terminator**	*ADH1* Term	*ADH1* Term	*ADH1* Term	*ADH1* Term
***E. coli* selection marker**	Ampicillin	Ampicillin	Ampicillin	Ampicillin
**Yeast auxotrophic selection marker**	*LEU2*	*LEU2*	*TRP1*	*TRP1*

Supplementary Table 6 | Yeast destination vectors.

**Forward**	**AD**	**SWIM**	5'-AGACGTGTGCTCTTCCGATCTNNNNNNNNNNNNNCGATGATGAAGATACCCCACCA-3′
		**Universal**	5'-CGCGTTTGGAATCACTACAGGG-3′
	**DB**	**SWIM**	5'-AGACGTGTGCTCTTCCGATCTNNNNNNNNNNNNNGGTCAAAGACAGTTGACTGTATCGT-3′
		**Universal**	5'-GGCTTCAGTGGAGACTGATATGCCTC-3′
**Reverse**	**AD**	**SWIM**	5'-GGAGACTTGACCAAACCTCTGGCG-3′
		**Universal**	5'-GGAGACTTGACCAAACCTCTGGCG-3′
	**DB**	**SWIM**	5'-GGAGACTTGACCAAACCTCTGGCG-3′
		**Universal**	5'-GGAGACTTGACCAAACCTCTGGCG-3′

Supplementary Table 7 | Primers used (Ns denotes 13-mer well index).

Assay version	DB vector	AD vector	DB yeast strain	AD yeast strain
4	pDEST-QZ212	pDEST-QZ213	Y8930	Y8800
1	pDEST-DB	pDEST-AD-*CYH2*	Y8930	Y8800

Supplementary Table 8 | Y2H version details.

Supplementary Note 1: Comprehensive assessment of existing protein interaction datasets

We systematically evaluated the quality of major yeast interaction datasets representing the full range of different mapping approaches (Supplementary Data 5-9). Specifically, the following datasets were experimentally evaluated: experimental structures (I3D-exp-17)[2]; literature-curated complexes (CYC2008)[3]; systematic proteome-scale datasets generated using: Y2H (Y2H-union-08: Uetz-screen, Ito-core, and CCSB-YI1)[4–6]; dihydrofolate reductase protein-fragment complementation assay (DHFR PCA)[7]; and AP/MS datasets (Gavin a/b, Ho, Krogan)[8–11]. Literature-curated interaction pairs[12–15] were divided into three subsets: (i) those found using only methods detecting co-complex associations (Lit-CC-17); (ii) those with a single report using a method detecting binary interactions (Lit-BS-17); and (iii) those with multiple evidence including a method detecting binary interactions (Lit-BM-17). Finally, we also tested protein interaction predictions using integrative Bayesian approaches (Jansen, PrePPI)[16,17] and functional associations from a genetic interaction profile similarity network (GI-PSN)[18].

To assess the accuracy of the datasets, we compared the recovery rates of representative samples of these datasets in up to three different assays benchmarked against scPRS-v2 and scRRS-v2. As described for I3D-exp-17 above, we tested all protein pairs for Lit-BM-17 and Y2H-union-08 using Y2H-v4, so that altogether 8,999 pairs were tested in this experiment (Fig. 1f, Supplementary Fig. 1g, and Supplementary Data 10). Moreover, since most of the systematic binary approaches and many of the studies that comprise Lit-BM made use of the Y2H assay, we also tested random samples of those datasets using two additional orthogonal assays, GPCA and MAPPIT (Fig. 1g, h, Supplementary Figs. 1h, i and Supplementary Data 11).

The recovery rates of I3D-exp-17, Lit-BM-17, and Y2H-union-08 approached the assay sensitivity limit, with the rate for Y2H-union-08 being indistinguishable from that of scPRS-v2 (P = 0.2, one-sided Fisher's exact test). Y2H-union-08 and I3D-exp-17 performed slightly but significantly better than Lit-BM-17 (P = 0.006, two-sided Fisher's exact test, Fig. 1f), a pattern that was confirmed in the GPCA and MAPPIT results for Lit-BM-13.

As shown previously[6,19], pairs supported by only a single piece of evidence in the literature (Lit-BS-17) were statistically indistinguishable from scRRS-v2 (Fig. 1f, P = 0.1, one-sided Fisher's exact test), demonstrating an extremely low level of quality for these pairs. Interestingly, the proteome-scale DHFR PCA dataset, reported to detect physically proximal but not necessarily directly-contacting protein pairs[7], had low recovery rates of 3% and 2%, in Y2H-v4 and MAPPIT respectively, but a higher recovery rate of 11% in GPCA (Fig. 1f-h). Pairs from AP/MS co-complex association datasets were also detected at lower rates than both scPRS-v2 and binary PPI datasets, but at significantly higher rates than the negative control scRRS-v2 (median P = 0.018, one-sided Fisher's exact test, Supplementary Fig. 1g). Again, this is consistent with the known high proportion of indirect associations in complexes[8–11].

The computational PPI predictions, PrePPI and Jansen[16,17], tested positive at low levels of 4% and 2%, respectively (Supplementary Fig. 1g) suggesting a low proportion of directly contacting protein pairs. For the GI-PSN[18] pairs, the GPCA recovery rate increased proportionally to the Pearson's correlation coefficient (PCC) threshold; the trend among the corresponding recovery rates in Y2H-v4 was more stable but consistent (Supplementary Fig. 1g, h, j). For the lower PCC thresholds of 0.2 and 0.3, the recovery rate of GI-PSN pairs in both assays was similar to that of manually-curated complexes (CYC2008). The GI-PSN results are consistent with the conclusions that: i) genes encoding proteins in complexes often share highly correlated genetic

interaction patterns[18]; and (ii) the pairs with the highest PCC more likely encode direct interaction partners[20]. Importantly, at PCC ≥ 0.5, where the direct binary PPI content substantially exceeds that of protein complexes, the GI-PSN contains only 841 edges (Supplementary Fig. 1k).

Although binary PPI assays tend to capture proteins in direct contact[21], the assays used in this study are performed in living cells and so endogenously-expressed proteins could bridge interactions between the tested pairs. To more precisely assess each experimental approach's ability to capture direct PPIs, and thus map the contactome, we explored the subsets of pairs within complexes with at least three different subunits (Supplementary Data 12). We found that Y2H-union-08 and Lit-BM-24 contained a high proportion of direct interactions, whereas AP/MS-06[8,9,11], had only a slight enrichment of direct PPIs over indirect associations (Supplementary Figs. 2a, b). GI-PSN captured a similar ratio of direct and indirect interactions as AP/MS-06, although the fraction of direct contacts increased with PCC value. Since the number of interactors that can simultaneously contact a given protein is fundamentally limited by the shape and surface area of the protein, the number of direct interactions within a complex scaled roughly linearly with its size, gaining an average of 3 PPIs per additional subunit. In contrast, the number of indirect associations scales quadratically and, as a result, the difference between the two is most dramatic for larger complexes (Supplementary Fig. 2c). Consequently, co-complex datasets will overrepresent the largest complexes relative to their true proportion of directly contacting PPIs in the interactome.

Supplementary Note 2: Guilt by association functional predictions with Y2H-union-25

Using a guilt-by-association approach[21] based on the GO term annotations of interaction partners in Y2H-union-25, we developed hypotheses for the functions of uncharacterised proteins within our PPI network (Supplementary Data 18). The lag between publications describing gene functions and their curation into GO terms sometimes yields genes that appear as “genes of unknown function”, even though their functions have been described. There are four such examples, which can be used to test the accuracy of our predictions. Firstly, owing to its recently demonstrated role as a regulator of peroxisomal abundance, *YGR168C* is now known as *PEX35*[22]. Ygr168c/Pex35 has 23 PPIs in Y2H-union-25, all from YeRI, out of which eight are proteins involved in peroxisomal biology, and so was predicted to function in peroxisomal protein import, showcasing the ability of Y2H-union-25 to predict gene function accurately (Supplementary Fig. 4i). Secondly, the highest ranked prediction for Yjr011c was to be involved in the CCR4-NOT complex and it is now known as Cal4, an accessory component of the CCR4-NOT complex[23]. Thirdly, the highest ranked prediction for Ykl075c is for mitochondrial respiratory chain complex II assembly. This gene is now named AAN1, based on “impacts on actin cable stability, mitochondrial function, BCAA metabolism, and cellular lifespan”, with its deletion being found to significantly reduce mitochondrial oxidation levels[24]. Ykl075c also localizes to the mitochondria upon rapamycin treatment[25]. Finally, Ypr174c is predicted to be localized to a membrane. It is now named Csa1 and found to anchor Cdc5 at spindle pole bodies[26]. It binds phosphatidylinositols and phosphatidylethanolamines[27] and is localized to the nuclear periphery[28]. In summary, where there is evidence to evaluate our predictions, it broadly supports their accuracy, especially considering the heuristic nature of their generation, and thus supports the useful information contained within our systematically generated PPI network.

Supplementary References

1. Zhang, J. *et al.* Predicting protein-protein interactions in the human proteome. *Science* **390**, eadt1630 (2025).
2. Mosca, R., Céol, A. & Aloy, P. Interactome3D: adding structural details to protein networks. *Nat. Methods* **10**, 47–53 (2013).
3. Pu, S., Wong, J., Turner, B., Cho, E. & Wodak, S. J. Up-to-date catalogues of yeast protein complexes. *Nucleic Acids Res.* **37**, 825–831 (2009).
4. Uetz, P. *et al.* A comprehensive analysis of protein–protein interactions in *Saccharomyces cerevisiae*. *Nature* **403**, 623–627 (2000).
5. Ito, T. *et al.* A comprehensive two-hybrid analysis to explore the yeast protein interactome. *PNAS* **98**, 4569–4574 (2001).
6. Yu, H. *et al.* High-Quality Binary Protein Interaction Map of the Yeast Interactome Network. *Science* **322**, 104–110 (2008).
7. Tarassov, K. *et al.* An *in vivo* map of the yeast protein interactome. *Science* **320**, 1465–1470 (2008).
8. Gavin, A.-C. *et al.* Functional organization of the yeast proteome by systematic analysis of protein complexes. *Nature* **415**, 141–147 (2002).
9. Gavin, A.-C. *et al.* Proteome survey reveals modularity of the yeast cell machinery. *Nature* **440**, 631–636 (2006).
10. Ho, Y. *et al.* Systematic identification of protein complexes in *Saccharomyces cerevisiae* by mass spectrometry. *Nature* **415**, 180–183 (2002).
11. Krogan, N. J. *et al.* Global landscape of protein complexes in the yeast *Saccharomyces cerevisiae*. *Nature* **440**, 637–643 (2006).
12. Orchard, S. *et al.* The MIntAct project–IntAct as a common curation platform for 11 molecular interaction databases. *Nucleic Acids Res.* **42**, D358–D363 (2014).
13. Chatr-aryamontri, A. *et al.* The BioGRID interaction database: 2017 update. *Nucleic Acids Res.* **45**, D369–D379 (2017).

14. Salwinski, L. *et al.* The Database of Interacting Proteins: 2004 update. *Nucleic Acids Res.* **32**, 449D–451 (2004).

15. Licata, L. *et al.* MINT, the molecular interaction database: 2012 update. *Nucleic Acids Res.* **40**, D857–D861 (2012).

16. Zhang, Q. C., Petrey, D., Garzón, J. I., Deng, L. & Honig, B. PrePPI: a structure-informed database of protein-protein interactions. *Nucleic Acids Res.* **41**, D828–D833 (2013).

17. Jansen, R. *et al.* A Bayesian networks approach for predicting protein-protein interactions from genomic data. *Science* **302**, 449–453 (2003).

18. Costanzo, M. *et al.* A global genetic interaction network maps a wiring diagram of cellular function. *Science* **353**, aaf1420 (2016).

19. Rolland, T. *et al.* A proteome-scale map of the human interactome network. *Cell* **159**, 1212–1226 (2014).

20. Meldal, B. H. M. *et al.* Analysing the yeast complexome-the Complex Portal rising to the challenge. *Nucleic Acids Res.* **49**, 3156–3167 (2021).

21. Luck, K. *et al.* A reference map of the human binary protein interactome. *Nature* **580**, 402–408 (2020).

22. Yofe, I. *et al.* Pex35 is a regulator of peroxisome abundance. *J. Cell Sci.* **130**, 791–804 (2017).

23. Pillet, B. *et al.* Dedicated chaperones coordinate co-translational regulation of ribosomal protein production with ribosome assembly to preserve proteostasis. *Elife* **11**, (2022).

24. Sing, C. N. *et al.* Identification of a modulator of the actin cytoskeleton, mitochondria, nutrient metabolism and lifespan in yeast. *Nat. Commun.* **13**, 2706 (2022).

25. Koh, J. L. Y. *et al.* CYCLoPs: a comprehensive database constructed from automated analysis of protein abundance and subcellular localization patterns in *Saccharomyces cerevisiae*. *G3* **5**, 1223–1232 (2015).

26. Örd, M. *et al.* Proline-rich motifs control G2-CDK target phosphorylation and priming an anchoring protein for Polo kinase localization. *Cell Rep.* **31**, 107757 (2020).

27. Gallego, O. *et al.* A systematic screen for protein-lipid interactions in *Saccharomyces cerevisiae*. *Mol. Syst. Biol.* **6**, 430 (2010).

28. Huh, W.-K. *et al.* Global analysis of protein localization in budding yeast. *Nature* **425**, 686–691 (2003).
