# Supplementary material for: Experimental assessment of AI-based interactome mapping
Source: Nat Commun. 2026 Apr 4;17:4894. doi: 10.1038/s41467-026-70942-x (PMC13230783; doi:10.1038/s41467-026-70942-x)
Supplement: Supplementary file 25 — Reporting Summary [file 41467_2026_70942_MOESM25_ESM.pdf]

nature portfolio

Corresponding author(s): Pascal Falter-Braun, David E. Hill, Michael A. Calderwood, Jean-Claude Twizere, Marc Vidal

Last updated by author(s): Dec 24, 2025

Reporting Summary

Nature Portfolio wishes to improve the reproducibility of the work that we publish. This form provides structure for consistency and transparency in reporting. For further information on Nature Portfolio policies, see our Editorial Policies and the Editorial Policy Checklist.

Statistics

For all statistical analyses, confirm that the following items are present in the figure legend, table legend, main text, or Methods section.

n/a	Confirmed	
☐	☒	The exact sample size (n) for each experimental group/condition, given as a discrete number and unit of measurement
☒	☐	A statement on whether measurements were taken from distinct samples or whether the same sample was measured repeatedly
☐	☒	The statistical test(s) used AND whether they are one- or two-sided<br>*Only common tests should be described solely by name; describe more complex techniques in the Methods section.*
☒	☐	A description of all covariates tested
☒	☐	A description of any assumptions or corrections, such as tests of normality and adjustment for multiple comparisons
☐	☒	A full description of the statistical parameters including central tendency (e.g. means) or other basic estimates (e.g. regression coefficient) AND variation (e.g. standard deviation) or associated estimates of uncertainty (e.g. confidence intervals)
☐	☒	For null hypothesis testing, the test statistic (e.g. F, t, r) with confidence intervals, effect sizes, degrees of freedom and P value noted<br>*Give P values as exact values whenever suitable.*
☐	☒	For Bayesian analysis, information on the choice of priors and Markov chain Monte Carlo settings
☒	☐	For hierarchical and complex designs, identification of the appropriate level for tests and full reporting of outcomes
☐	☒	Estimates of effect sizes (e.g. Cohen's d, Pearson's r), indicating how they were calculated

Our web collection on statistics for biologists contains articles on many of the points above.

Software and code

Policy information about availability of computer code

Data collection	Bowtie2 2.2.3<br>samtools 1.2<br>Illumina bcl2fastq 2.20<br>BLAT 3.6.x1<br>Python 2.7.10<br>MySQL 5.6.26<br>Custom code for Y2H screening pipeline<br>Colonyzer 2
Data analysis	https://github.com/ccsb-dfci/ai-interactome-experimental-assessment<br>Cytoscape 3.8.2<br>Python 3.8.12<br>numpy 1.23.0<br>scipy 1.8.1<br>pandas 1.4.3<br>matplotlib 3.5.2<br>ipython 8.4.0<br>jupyter 1.0.0<br>notebook 6.4.12

seaborn 0.11.2<br>AlphaFold 2.3.1

For manuscripts utilizing custom algorithms or software that are central to the research but not yet described in published literature, software must be made available to editors and reviewers. We strongly encourage code deposition in a community repository (e.g. GitHub). See the Nature Portfolio guidelines for submitting code & software for further information.

Data

Policy information about availability of data

All manuscripts must include a data availability statement. This statement should provide the following information, where applicable:

- Accession codes, unique identifiers, or web links for publicly available datasets
- A description of any restrictions on data availability
- For clinical datasets or third party data, please ensure that the statement adheres to our policy

The data is available in the supplementary data tables. Protein interaction data have been submitted to the IMEx consortium through IntAct and assigned the identifier IM-30553 [https://imexcentral.org/icentral/pubedit.action?id=30553]. Predicted structures of YeRI PPIs are deposited at doi:10.5281/zenodo.18601049 [https://zenodo.org/records/18601049]. YeRI, Y2H-union-25, and ValBin-25 maps are available at our website: https://yeast.interactome-atlas.org/. Analysis code is archived together with the input data at doi:10.5281/zenodo.18499797 [https://zenodo.org/records/18499797].

Research involving human participants, their data, or biological material

Policy information about studies with human participants or human data. See also policy information about sex, gender (identity/presentation), and sexual orientation and race, ethnicity and racism.

Reporting on sex and gender	N/A
Reporting on race, ethnicity, or other socially relevant groupings	N/A
Population characteristics	N/A
Recruitment	N/A
Ethics oversight	N/A

Note that full information on the approval of the study protocol must also be provided in the manuscript.

Field-specific reporting

Please select the one below that is the best fit for your research. If you are not sure, read the appropriate sections before making your selection.

☒ Life sciences ☐ Behavioural & social sciences ☐ Ecological, evolutionary & environmental sciences

For a reference copy of the document with all sections, see nature.com/documents/nr-reporting-summary-flat.pdf

Life sciences study design

All studies must disclose on these points even when the disclosure is negative.

Sample size	For PPI assays where a random sample of a dataset was used, a size of ~250 pairs was chosen based on a 95% CI value of 5% from inputing an recovery rate of 20% using the standard error of proportion. One exception to this is the test of the human predicted PPIs, where we chose a sample size of ~4,000 pairs, which was the largest number we could test in a single experimental batch.
Data exclusions	No data were excluded.
Replication	*Describe the measures taken to verify the reproducibility of the experimental findings. If all attempts at replication were successful, confirm this OR if there are any findings that were not replicated or cannot be reproduced, note this and describe why.*
Randomization	Screen pairs and controls for Y2H v4 pairwise test, MAPPIT and GPCA were randomly arranged on well plates.
Blinding	Manual scoring of Y2H pairwise tests was blinded.

Reporting for specific materials, systems and methods

We require information from authors about some types of materials, experimental systems and methods used in many studies. Here, indicate whether each material, system or method listed is relevant to your study. If you are not sure if a list item applies to your research, read the appropriate section before selecting a response.

Materials & experimental systems

n/a	Involved in the study	
☒	☐	Antibodies
☐	☒	Eukaryotic cell lines
☒	☐	Palaeontology and archaeology
☒	☐	Animals and other organisms
☒	☐	Clinical data
☒	☐	Dual use research of concern
☒	☐	Plants

Methods

n/a	Involved in the study	
☒	☐	ChIP-seq
☒	☐	Flow cytometry
☒	☐	MRI-based neuroimaging

Eukaryotic cell lines

Policy information about cell lines and Sex and Gender in Research

Cell line source(s)	1. Yeast strains - Y8800 and Y8930: the same strain used in many previous studies (Rolland et al. Cell. 2014 Nov 20;159 (5);5:4767.)<br>2.HEK293T for GPCA: the same cell line used in previous studies (Choi et al. Nat Commun. 2019 Aug 29;10(1):3907.)<br>3. HEK293T for MAPPIT: it was obtained by our department in 1996 from Dr, Mark Hall at the Biochemistry department of the University of Birmingham.
Authentication	All cell lines except HEK293T for MAPPIT were not re-authenticated.<br>HEK293T for MAPPIT was authenticated by analyzing single nucleotide polymorphism (Lin et al. Nat Commun. 2014 Sep 3;5:4767.)
Mycoplasma contamination	All the cell lines were tested with DNA stain for mycoplasma contamination and were free of contamination
Commonly misidentified lines (See ICLAC register)	No commonly misidentified lines were used.

Plants

Seed stocks	*Report on the source of all seed stocks or other plant material used. If applicable, state the seed stock centre and catalogue number. If plant specimens were collected from the field, describe the collection location, date and sampling procedures.*
Novel plant genotypes	*Describe the methods by which all novel plant genotypes were produced. This includes those generated by transgenic approaches, gene editing, chemical/radiation-based mutagenesis and hybridization. For transgenic lines, describe the transformation method, the number of independent lines analyzed and the generation upon which experiments were performed. For gene-edited lines, describe the editor used, the endogenous sequence targeted for editing, the targeting guide RNA sequence (if applicable) and how the editor was applied.*
Authentication	*Describe any authentication procedures for each seed stock used or novel genotype generated. Describe any experiments used to assess the effect of a mutation and, where applicable, how potential secondary effects (e.g. second site T-DNA insertions, mosiacism, off-target gene editing) were examined.*
